# Anticoagulant rodenticide exposure and toxicosis in bald eagles (*Haliaeetus leucocephalus*) and golden eagles (*Aquila chrysaetos*) in the United States

**DOI:** 10.1371/journal.pone.0246134

**Published:** 2021-04-07

**Authors:** Kevin D. Niedringhaus, Nicole M. Nemeth, Samantha Gibbs, Jared Zimmerman, Lisa Shender, Kate Slankard, Heather Fenton, Bahnson Charlie, Martha Frances Dalton, Elizabeth J. Elsmo, Robert Poppenga, Brian Millsap, Mark G. Ruder

**Affiliations:** 1 Southeastern Cooperative Wildlife Disease Study, College of Veterinary Medicine, University of Georgia, Athens, GA, United States of America; 2 United States Fish and Wildlife Service, National Wildlife Refuge System, Chiefland, FL, United States of America; 3 Florida Fish and Wildlife Conservation Commission, Gainesville, FL, United States of America; 4 Kentucky Department of Fish and Wildlife Resources, Frankfort, KY, United States of America; 5 California Animal Health and Food Safety Laboratories, School of Veterinary Medicine, University of California, Davis, CA, United States of America; 6 United States Fish and Wildlife Service, Division of Migratory Bird Management, Albuquerque, New MX, United States of America; Universidade Federal de Minas Gerais, BRAZIL

## Abstract

Raptors, including eagles, are geographically widespread and sit atop the food chain, thereby serving an important role in maintaining ecosystem balance. After facing population declines associated with exposure to organochlorine insecticides such as dichlorodiphenyltrichloroethane (DDT), bald eagles (*Haliaeetus leucocephalus*) have recovered from the brink of extinction. However, both bald and golden eagles (*Aquila chrysaetos*) are exposed to a variety of other toxic compounds in the environment that could have population impacts. Few studies have focused on anticoagulant rodenticide (AR) exposure in eagles. Therefore, the purpose of this study was to determine the types of ARs that eagles are exposed to in the USA and better define the extent of toxicosis (i.e., fatal illness due to compound exposure). Diagnostic case records from bald and golden eagles submitted to the Southeastern Cooperative Wildlife Disease Study (University of Georgia) 2014 through 2018 were reviewed. Overall, 303 eagles were examined, and the livers from 116 bald eagles and 17 golden eagles were tested for ARs. The percentage of AR exposure (i.e., detectable levels but not associated with mortality) in eagles was high; ARs were detected in 109 (82%) eagles, including 96 (83%) bald eagles and 13 (77%) golden eagles. Anticoagulant rodenticide toxicosis was determined to be the cause of mortality in 12 (4%) of the 303 eagles examined, including 11 bald eagles and 1 golden eagle. Six different AR compounds were detected in these eagles, with brodifacoum and bromadiolone most frequently detected (81% and 25% of eagles tested, respectively). These results suggest that some ARs, most notably brodifacoum, are widespread in the environment and are commonly consumed by eagles. This highlights the need for research to understand the pathways of AR exposure in eagles, which may help inform policy and regulatory actions to mitigate AR exposure risk.

## Introduction

While golden eagle (*Aquila chrysaetos*) populations have remained relatively stable since the 1970s, bald eagle (*Haliaeetus leucocephalus*) populations have made dramatic recoveries since the banning of dichlorodiphenyltrichloroethane (DDT) in 1972 [1,2]. However, many threats from other toxicants still exist for eagles and other raptors, including exposure to anticoagulant rodenticides (ARs; [3–5]). Anticoagulant rodenticides are used globally to control rodents in urban and suburban settings, as well as in agricultural and conservation or habitat restoration settings [6]. First and second generation anticoagulant rodenticides (FGARs and SGARs, respectively) interfere with the activation of vitamin K-dependent clotting factors in the liver, which may lead to life-threatening hemorrhage following minor trauma or exertion during routine activities [6,7]. The two groups of chemicals cause similar clinical signs in intoxicated animals. However, compared with FGARs, SGARs have a longer half-life in the tissues and a lower LD_50_ in multiple domesticated animals (summarized by [8]) resulting in greater potential for intoxication after a single ingestion [4,7]. These properties make SGARs more efficient at killing target rodent species, but also increase the risk of non-target wildlife intoxication through both primary and secondary exposures [4]. In 2008, the United States Environmental Protection Agency (US EPA) published a final risk mitigation decision on ten rodenticide compounds aimed, in part, to reduce wildlife exposures to SGARs [9]. This, and subsequent actions by the EPA, placed limits on the sale and distribution of products containing SGARs to general consumers and placed regulations on products for use by pest management professionals during structural pest control activities or for agricultural applications [9,10]. Despite these risk mitigation actions, AR exposure is common in numerous wildlife taxa [11,12].

Exposure to and resulting toxicosis due to ARs is well documented in numerous raptor species and typically occurs as secondary exposure after ingestion of exposed or intoxicated prey species, including rodents and other small mammals [13–16]. Individual case reports of mortality due to AR toxicosis, as well as larger subclinical exposure studies, have been described in multiple raptor species [8,17,18]. However, few studies have assessed the types of compounds and tissue concentrations in bald and golden eagles. Therefore, the purpose of this study was to determine the prevalence of exposure and toxicosis, geographic distribution, and types of ARs detected in the liver of bald and golden eagles that underwent diagnostic evaluation at the Southeastern Cooperative Wildlife Disease Study (SCWDS; University of Georgia) in the United States.

## Materials and methods

Fresh or frozen whole carcasses or select tissues collected from deceased bald and golden eagles were submitted to SCWDS by personnel from 18 supporting state wildlife management agencies and the United States Fish and Wildlife Service from 2014 through 2018. Specimens were submitted for morbidity and mortality investigations as part of each agency’s wildlife health program. All birds were aged according to feather coloration on the head and body and were categorized as first-year, immature (one to four years) or adult (≥ five years). Complete postmortem examinations were performed on all eagles, including gross and histopathologic examination, as well as additional ancillary diagnostic testing as needed to arrive at a diagnosis. Ancillary tests included radiology, bacteriology, virology, parasitology, and toxicology. Additionally, sex, county and state of origin were recorded for each bird. Upon completion of diagnostic investigations, eagle carcasses were transferred to the National Eagle Repository (Commerce City, Colorado).

Liver samples from a subset of submitted eagles (n = 133) were submitted to the California Animal Health and Food Safety Laboratories (CAHFSL; Davis, California) for quantitative testing of ARs by reverse phase ultraperformance liquid chromatography-tandem mass spectrometry [19]. Liver tissue was submitted from all eagles with lesions consistent with AR toxicosis (i.e., unexplained hemorrhage), as well as from a subset presumed to have died from other causes that were selected randomly or at the request of the original submitter. The assay detects the SGARs brodifacoum, bromadiolone, difenacoum, and difethialone, as well as the FGARs chlorophacinone, diphacinone, coumachlor, and warfarin. Detection limits spanned 0.75–25 ng/g wet weight, and lower quantitation limits were established as 50 ng/g. Trace levels were reported when a compound was detected but was below a quantifiable level. The quantity and type of each AR detected, if any, were recorded for each bird. Any eagle with detected levels of one or more ARs, regardless of concentration, was categorized as ‘exposed.’ Diagnosis of ‘AR toxicosis’ required detection of at least one AR compound in the liver in addition to gross or microscopic evidence of hemorrhage anywhere in the carcass (e.g., subcutaneous, intramuscular, intracoelomic, visceral) determined to be unrelated to other potential causes (e.g., trauma or infection). Traumatic causes of mortality (e.g., gunshot, collision, intraspecific aggression, electrocution, unknown) in eagles were diagnosed based on presence of consistent postmortem lesions. All examinations were performed by licensed veterinarians with additional training or specialized qualifications in veterinary pathology.

## Results

Between 2014 and 2018, 247 bald eagles and 56 golden eagles were submitted to SCWDS for diagnostic evaluation. Golden eagle submissions were primarily from states in the western USA: AK (n = 1), AZ (n = 1), CO (n = 5), GA (n = 2), ID (n = 5), KS (n = 1), MT (n = 1), NE (n = 3), NM (n = 10), NV (n = 8), OK (n = 1), OR (n = 1), PA (n = 2), TX (n = 3), UT (n = 5), WA (n = 2), WV(n = 1), and WY (n = 4). Bald eagle submissions were primarily from states in the eastern USA: AL (n = 1), AR (n = 3), FL (n = 60), GA (n = 35), KY (n = 15), LA (n = 2), MD (n = 1), MO (n = 1), MS (n = 1), NC (n = 6), PA (n = 104), SC (n = 4), TN (n = 1), UT (n = 3), VA (n = 2), and WV (n = 7). Tissues from 116/247 (47%) bald eagles and 17/56 (30%) golden eagles totaling 133 eagles (44% of all eagles) were tested for ARs. Table 1 provides a summary of overall AR exposure among bald eagles and golden eagles, as well as a breakdown of birds exposed to a single AR, or more than one AR.

**Table 1:** Percent exposure and number of anticoagulant rodenticide (AR) compounds detected in bald and golden eagles submitted to the Southeastern Cooperative Wildlife Disease Study (University of Georgia), 2014–2018.

		Overall AR exposure		1 AR	>1 AR
	No. tested	No. exposed	% exposed	No. exposed (%)	No. exposed (%)
**Bald eagles**	116	96	83	56 (48)	40 (35)
**Golden eagles**	17	13	77	4 (24)	9 (53)
**Overall**	133	109	82	60 (45)	49 (37)

A summary of the quantity and types of ARs detected is presented in Table 2. The most commonly detected compound in all eagles tested was brodifacoum (107/133; 81%). Coumachlor and warfarin, two of the FGARs included in the testing panel, were not detected in tissues from any eagle.

**Table 2:** The frequency of eight anticoagulant rodenticide (AR) compounds detected in 116 bald eagles and 17 golden eagles submitted to the Southeastern Cooperative Wildlife Disease Study (University of Georgia), 2014–2018.

AR Compound	Bald eagles exposed/116 tested (%)	Golden eagles exposed/17 tested (%)	Total exposed/133 tested (%)
First Generation AR			
Chlorophacinone	1 (1)	1 (6)	2 (2)
Coumachlor	0 (0)	0 (0)	0 (0)
Diphacinone	1 (1)	2 (12)	3 (2.3)
Warfarin	0 (0)	0 (0)	0 (0)
Second Generation AR			
Brodifacoum	98 (85)	1 (65)	107 (81)
Difenacoum	11* (10)	2 (12)	13* (10)
Difethialone	21 (18)	1 (6)	22 (17)
Bromadiolone	25 (22)	8 (47)	33 (25)

*Testing panels in two bald eagles in 2014 did not include difenacoum (n = 114 bald eagles, 131 total eagles).

**Fig 1:**
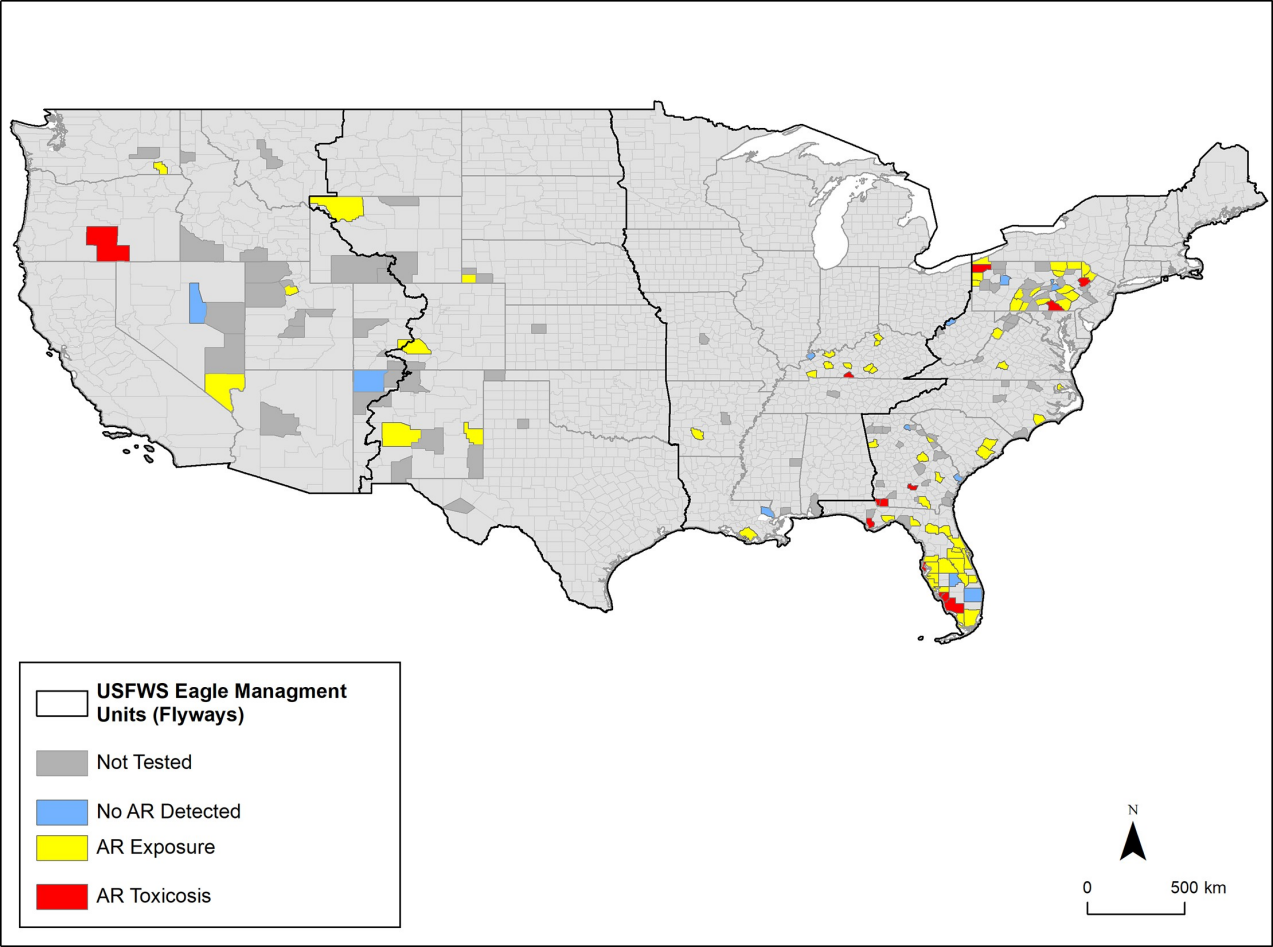
The geographic distribution of eagles submitted for postmortem examination to the Southeastern Cooperative Wildlife Disease Study, 2014–2018. Highlighted are counties dead eagles were received; and which eagles were not tested for AR (dark gray), no AR was detected (blue), AR exposure documented (yellow), or AR toxicosis confirmed (red). One eagle submitted from Matanuska Susitna County, Alaska is not shown and had no ARs detected. Black lines represent United States Fish and Wildlife Service Eagle Management Units, designated by the United States Migratory Bird Flyways.

The geographic distribution of eagles with AR exposure and toxicosis is depicted in Fig 1.

**Fig 2:**
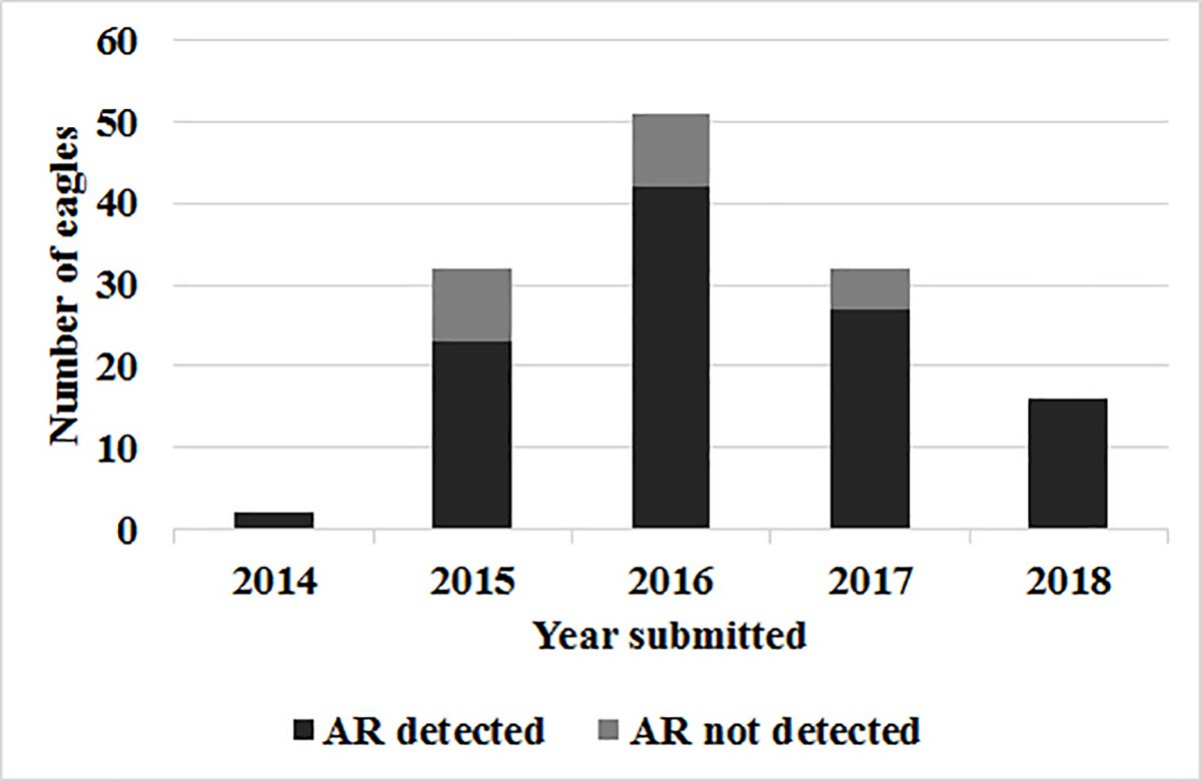
Bar graph depicting the number of eagles submitted to the Southeastern Cooperative Wildlife Disease Study, 2014–2018, that were tested for anticoagulant rodenticides and those in which one or more AR compounds were detected.

The highest numbers of eagles were submitted by the states of Pennsylvania (106 eagles; 35%), Florida (59 eagles; 20%), and Georgia (37 eagles; 12%). Within these three states, ARs were detected in 29/35 (83%) eagles tested from Pennsylvania, 44/52 (85%) eagles tested from Florida, and 9/11 (82%) eagles tested from Georgia. During the study, eagles were most commonly submitted and tested for ARs in 2016 (51) followed by 2017 and 2015 (32 eagles each year). Fig 2 shows the distribution of eagles tested for and exposed to ARs per study year.

Anticoagulant rodenticide toxicosis was diagnosed as the cause of mortality in 11/247 (5%) bald eagles examined and 1/56 (2%) golden eagles examined, which represents 4% (12/303) of all eagles examined. Seven eagles (6 bald eagles, 1 golden eagle) that died from AR toxicosis had multiple compounds detected. Among birds diagnosed with AR toxicosis, brodifacoum was detected in 100% (12/12) of eagles. Difethialone was detected in 5/12 (42%) eagles diagnosed with AR toxicosis, bromadiolone in 4/12 (33%) eagles, and difenacoum in 1/12 (8%) eagle. The single golden eagle diagnosed with AR intoxication had brodifacoum and bromadiolone detected in its tissues. No FGARs, including chlorophacinone, coumachlor, diphacinone, or warfarin, were detected among eagles that died from AR toxicosis; all 12 eagle deaths were associated exclusively with SGARs.

**Fig 3:**
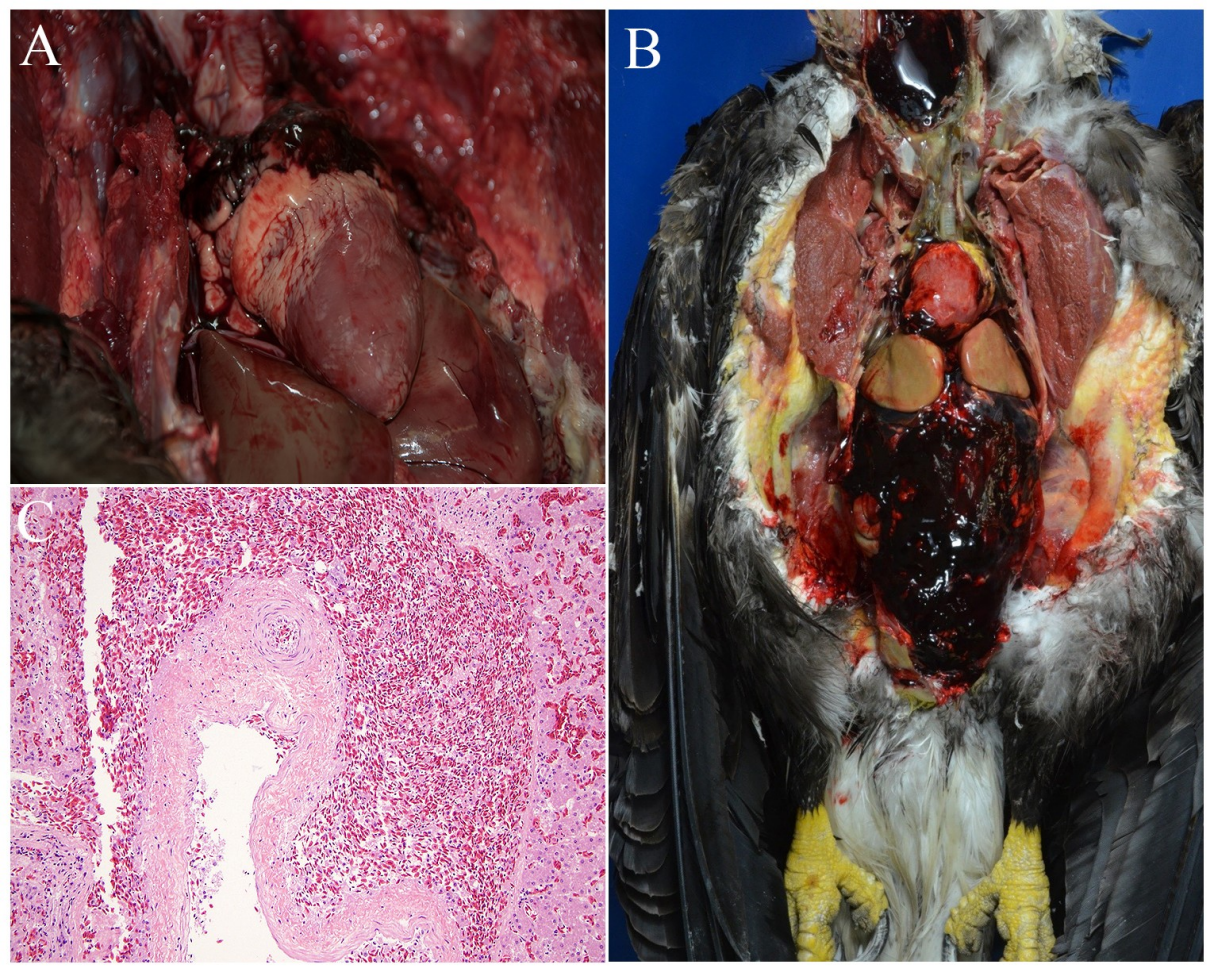
Lesions associated with AR toxicosis in eagles. A: A bald eagle with unclotted blood surrounding the base of the heart within the coelom. B: A bald eagle with abundant, unclotted blood overlying the liver and surrounding the heart and filling much of the coelomic cavity. C: Photomicrograph of the liver from a bald eagle showing abundant erythrocytes (hemorrhage) outside an arterial wall and spilling into the adjacent parenchyma. Hematoxylin and eosin (HE) stain.

Among eagles diagnosed with AR toxicosis, the grossly evident (i.e., anatomic) and histologic distribution of the associated hemorrhage most commonly included the coelomic cavity (9/12; 75%), gastrointestinal tract (6/12; 50%), and lungs (4/12; 33%; Fig 3). A summary of the compounds detected, distribution of lesions, and demographic and geographic information on the 12 cases of confirmed AR toxicosis is presented in Table 3.

**Table 3:** Geographic, demographic, anticoagulant rodenticide (AR) compound liver levels, and anatomical location of hemorrhage for bald eagles (BAEA) and golden eagles (GOEA) diagnosed with AR toxicosis that were submitted to the Southeastern Cooperative Wildlife Disease Study (University of Georgia), 2014–2018.

Case	Species	Location (County, State)	Year	Age	Sex	Anticoagulant rodenticide compound and concentration (ng/g wet weight)				Anatomic location of hemorrhage*
						Brodifacoum	Bromadiolone	Difethialone	Difenacoum	
1	BAEA	Irwin, GA	2014	Ad.	F	Trace	ND	ND	ND	Coelomic cavity, subcutis, intra-ocular
2	BAEA	Monroe, PA	2015	Ad.	F	Trace	ND	ND	ND	Oropharynx, pulmonary interstitium*, gastrointestinal tract, coelomic cavity
3	BAEA	Pinellas, FL	2016	Ad.	F	96	Trace	ND	ND	Lung, ovary, heart, brain*
4	BAEA	Crawford, PA	2016	Juv.	F	170	ND	ND	ND	Lung, coelomic cavity, heart, proventriculus
5	BAEA	Collier, FL	2016	Ad.	F	490	85	Trace	ND	Coelomic cavity, large intestinal lumen*, kidney*
6	BAEA	Gulf, FL	2017	Ad.	F	Trace	ND	ND	ND	Coelomic cavity, periarticular, pulmonary interstitium*, brain*, liver*
7	GOEA	Lake, OR	2017	Juv.	F	Trace	Trace	ND	ND	Coelomic cavity, perirenal adipose, pulmonary interstitium*, spleen
8	BAEA	Seminole, GA	2018	Ad.	F	Trace	Trace	Trace	ND	Oropharynx, coelomic cavity
9	BAEA	Allen, KY	2018	Ad.	F	77	ND	ND	ND	Intestinal and proventricular serosa, skeletal muscle
10	BAEA	Decatur, GA	2018	Ad.	M	Trace	ND	Trace	ND	Coelomic cavity
11	BAEA	Lee, FL	2018	Ad.	M	760	ND	Trace	Trace	Brain*, esophagus*, intestinal serosa, kidney*
12	BAEA	York, PA	2018	Ad	F	69	ND	Trace	ND	Coelomic cavity

* = histological lesion only.

Among all eagles (n = 303) submitted to SCWDS from 2014 to 2018, the most commonly diagnosed cause of mortality was trauma (e.g., collision, gunshot, electrocution, unknown); this included 49% (147/303) of all eagles, 47% (116/247) of bald eagles, and 55% (31/56) of golden eagles. Among bald eagles (n = 116) and golden eagles (n = 17) tested for ARs, 35 and four, respectively, were diagnosed with both trauma and AR exposure.

## Discussion

Our data indicate that among eagles submitted to a large wildlife research and diagnostic service and tested for AR compounds over a multi-year period, the majority were exposed to one or more AR compounds. Exposure rates were over 80% among eagles from the three states with the highest numbers of submissions (Pennsylvania, Florida, and Georgia). However, AR toxicosis was infrequently diagnosed as a primary cause of mortality in eagles. Despite the high prevalence of AR compounds detected in eagle tissues in the present study, approximately 4% of eagles were determined to have died from AR toxicosis. This may suggest that overt death due to ingestion of high AR quantities is relatively rare among these birds. Conversely, it is possible this is an underestimate because of biases and limitations associated with the passive detection of sick and dead wildlife [20] or difficulty in determining thresholds for toxicosis. Furthermore, much remains to be understood regarding individual and population-level implications of subclinical AR exposure and non-lethal effects in eagles, as well as in many other wildlife species [21].

The pathways of AR exposure in the bald and golden eagles in this study are not clear and such an understanding remains a significant research gap [22]. As predatory and scavenging species high in the trophic web, eagles are at risk for repeat exposure and bioaccumulation of ARs [5]. Compared to other raptors, bald eagles less commonly predate rodents and thus have probably been a less frequent target for studies documenting AR tissue residues. Interestingly, we recorded a similar prevalence of AR exposure in bald eagles when compared to golden eagles, which are known to regularly consume rodents and other mammals. Further study is warranted to understand the pathways of AR exposure in these two eagle species, as well as other raptors [5]. There have been relatively few studies to investigate AR exposure or toxicosis specifically in eagles; such data have typically been limited to a component of larger raptor toxicology or mortality studies. Retrospective studies investigating causes of eagle mortality identified AR toxicosis as an extremely rare and often non-existent cause of mortality in bald eagles in western Canada [23], Virginia [24] and throughout the USA [25]. One study described mortality in a bald eagle (in 1995) and a golden eagle (in 1996) in New York associated with warfarin and brodifacoum toxicosis, respectively, but eagles were under-represented in this study compared to other raptor species [8]. Samples from golden eagles in California in the late 1990’s revealed 8/10 (80%) eagles with brodifacoum exposure [26], and in New York in 1998–2001, 1/5 (20%) bald eagles had AR exposure [27]. The only other large study of AR exposure specifically in eagles was conducted in Norway. In that study, 69% of 16 golden eagles had detectable levels of SGARs, including brodifacoum, bromadiolone, difenacoum, and/or flocoumafen [28]. As resourceful predators and scavengers that inhabit diverse habitats that may include landscapes with variable human influence (e.g., agricultural lands, industrial and suburban development), bald and golden eagles are likely exposed to ARs through a variety of potentially complex, interacting, and variable pathways that are poorly understood but certainly extend beyond consumption of target rodent populations [22]. Investigations of potential ecological factors driving AR exposure are important for mitigating risk through effective policy and management [5,22].

Despite likely complex contributors to AR exposure and toxicosis across the landscape related to biological differences between species, the percentage of bald eagles (83%; 96/116 tested) and golden eagles (77%, 13/17 tested) with AR exposure in this study was comparable to those in other raptor species from previous studies in the USA. In Massachusetts from 2006–2010, 89% (n = 80) of red-tailed hawks (*Buteo jamaicensis*), 73% (n = 40) of barred owls (*Strix varia*), 87% (n = 23) of eastern screech owls (*Megascops asio*), and 100% (n = 18) of great-horned owls (*Bubo virginianus*) had detectable brodifacoum levels in the liver [17]. In New Jersey from 2008 to 2010, 81% (n = 105) of red-tailed hawks and 82% (n = 22) of great-horned owls had brodifacoum and/or bromadiolone detected within the liver [18]. Slankard and others (2019) [29] showed that 33% of 48 barn owls (*Tyto alba*) sampled in Kentucky from 2012–2016 had at least one AR detected in the liver. Stone et al. (2003) [27] investigated AR exposure in 19 raptor species in New York and observed the highest prevalence of AR exposure in great horned owls (81%; n = 53) and red-tailed hawks (58%, n = 78) among species with more than ten individuals tested. Studies in Europe showed a similar prevalence of AR exposure in raptors to those observed in the USA. In the United Kingdom, [30] showed that 84% of barn owls, 94% of red kites (*Milvus milvus*), and 100% of Eurasian kestrels (*Falco tinnunculus*) had detectable ARs in liver samples. In France, 3/4 (75%) Eurasian kestrels, 7/10 (70%) barn owls, 2/5 (20%) tawny owls (*Strix aluco*), and 10/11 (91%) common buzzards (*Buteo buteo*) had SGAR exposure as detected in liver samples [31]. Although varying study designs prevent direct comparisons across these studies, the high prevalence of AR exposure commonly reported in multiple raptor species is alarming.

Despite variation in the number of eagle submissions by states in the present study, we documented AR exposure in bald and/or golden eagles in all four United States Migratory Bird Flyways. Further, AR toxicosis was documented in all but the Central Flyway, including a golden eagle in the Pacific Flyway, and bald eagles in the Mississippi and Atlantic Flyways. Although variation in the geographic distribution of a variety of raptor species with AR exposure has been documented [15,32], we did not observe obvious patterns. However, there was significant variation in geographic distribution of case submissions by species with 91% (51/56) of golden eagles originating from the Pacific and Central Flyways and 99% (243/246) of bald eagles originating from the Mississippi and Atlantic Flyways. This may explain the observed, yet subtle variation in the types of compounds detected in tissues of bald and golden eagles (e.g., 47% of golden eagles were exposed to bromadiolone compared to 22% of bald eagles), as it relates to regional differences in availability of common prey species, toxicant use, agricultural practices, and land use. It is possible that targeted active surveillance for ARs among particular eagle populations (e.g., bald eagles in the western United States) could reveal meaningful patterns. Overall, we surmise the geographic distribution of exposure and toxicosis in the present study may relate to submission biases, rather than the true distribution or frequency of AR exposure on the landscape. This study was dependent upon reporting of moribund or dead eagles by the public, prompt recovery of carcasses, as well as submission of cases by wildlife agency personnel. The large number of submissions from the eastern USA is, in part, related to the geographic region of SCWDS member state wildlife agencies (i.e., predominantly the southeastern USA). Diagnostic data from wildlife often carries an inherent submission bias based on carcasses that are more easily seen and recovered (e.g., animal size and habitat type dependent) and motivated public or biologist response to report or submit them. Further, numerous golden eagles were submitted as part of satellite-GPS tracking and monitoring efforts by the USFWS, which allowed for improved carcass recovery.

Brodifacoum and bromadiolone were the most frequently detected AR compounds in eagles in this study. In particular, brodifacoum was detected in 81% of eagles (85% bald eagles, 65% golden eagles) tested for ARs and was found in 100% of the eagles diagnosed with AR toxicosis. The frequent detection of brodifacoum is similar to previous reports in barn, barred, and great horned owls in several regions of Canada and the eastern USA (Kentucky), multiple raptor species in the northeastern USA (New York), barn owls and tawny owls (United Kingdom), and golden eagles (California) [26,27,29,30,32]. The most commonly detected ARs in this study were SGARs, which may reflect their popularity as a rodenticide during the study period, but also their tendency to persist longer in tissues [7,32–34]. The prolonged tissue residues of SGARs, in particular brodifacoum, highlight the potential for multiple AR compound exposures in individual birds. For instance, of the 109 eagles (out of 133 tested) with AR exposure in this study, 40/96 (42%) bald eagles and 9/13 (69%) golden eagles were exposed to more than one AR compound and 58% (7/12) of eagles (1/1 golden eagle, 6/11 bald eagles) diagnosed with AR toxicosis were exposed to more than one AR compound. Results of controlled AR exposures reveal that SGAR-exposed American kestrels subsequently exposed to a FGAR may experience more adverse anticoagulant effects than were observed in naïve and previously FGAR-exposed kestrels [34]. Such experimental exposure studies, together with field evidence presented in this and other field studies, suggest an increased opportunity for bioaccumulation of one or more AR compounds and subsequent cumulative effects [5,34].

Although SGARs were commonly detected in the current study, the only FGARs we detected in bald and golden eagle tissues were chlorophacinone and diphacinone. This included only two bald eagles (n = 116 tested) and three golden eagles (n = 17 tested) (Table 2). The scarcity of detected FGARs among tested eagles may be related to lack of exposure and/or shorter AR compound half-lives in host tissues [32,35].

The diagnosis of AR toxicosis in raptors and other wildlife has numerous challenges [36]. The specific concentrations of individual AR compounds that cause coagulopathy in bald eagles and golden eagles have not been determined due to the inability to perform experimental studies to determine lethal doses. In the present study, some eagles had gross or microscopic evidence of hemorrhage consistent with AR toxicosis, with only trace levels of compounds detected. Conversely, other eagles had AR levels as high as 750 ng/g (brodifacoum) with no gross or microscopic evidence of coagulopathy, further emphasizing the diagnostic challenge in these cases. Additionally, the anatomic location and severity of observed hemorrhage are highly variable, and may be affected by concurrent diagnoses, such as trauma. In our study, we diagnosed trauma (e.g., collision, intraspecific aggression, gunshot, electrocution, unknown) as the cause of mortality in 49% (147/303) of eagles, including 47% (116/247) of bald eagles and 55% (31/56) of golden eagles. Among these cases of trauma in which AR testing occurred, 35 bald eagles and four golden eagles with traumatic injuries also had confirmed AR exposure. Confirming AR toxicosis in such cases is challenging, and it is possible that hemorrhage observed in some of these eagles was worsened by AR exposure. When live bird sampling is possible, the use of blood clotting assays (e.g., prothrombin time), in conjunction with other diagnostic tools, may assist in the diagnosis of AR exposure and intoxication in certain settings and is an area worthy of further study [37]. Additional diagnostic challenges are the marked variation in the postmortem quality of the samples and potential complications of freeze-thaw artifact, which can reduce the ability to detect microscopic lesions. As a result, wildlife biologists, diagnosticians and veterinarians may approach and interpret cases of suspected AR toxicosis differently, resulting in variable clinical and postmortem diagnoses.

Causes of wildlife mortality are often multifactorial, and as such, determining potential underlying risk factors and mechanism is challenging. Herring and others (2017) [5] have speculated that while exposure of golden eagles to lead, ARs, and physical trauma in the western USA may occur independently, the potential for synergistic effects should be explored [5]. Considering how common AR exposure was observed in both bald eagles and golden eagles in the current study, the potential for sublethal effects of AR exposure should be further studied to assess for more subtle effects on behavior, survivability, or reproduction as described in other species [38,39]. Thomas et al. (2011) [15] performed a probabilistic analysis to determine potentially significant AR levels in the liver of red-tailed hawks, barn owls, barred owls, and great-horned owls. This type of analysis would be useful with a larger dataset than ours, containing a higher sample size of eagles with AR toxicosis.

Results also reveal that despite the US EPAs mitigation efforts implemented for SGARs in 2015, there is still widespread exposure among bald and golden eagle populations to these compounds. Our study did not look for temporal change in the prevalence of exposure, and future AR studies on eagles should do so to determine if a decline in exposure results from recent regulatory changes. Slankard and others (2019) [29] examined prevalence of AR exposure in barn owls in Kentucky 2012 to 2016 and observed a decline in prevalence over the course of the study. It is unclear if the decline in SGAR exposure in barn owls was directly related to the 2015 implementation of US EPA mitigation efforts or if it represents species-specific variation or geographical differences in SGAR availability and use. Previous studies examining AR levels in eagles largely pre-dated the year 2015, and thus have limited utility toward assessing recent trends in AR exposure in eagles. The contributing factors to the high percentages of eagles exposed to AR compounds in the present study are unknown, but may relate to the continued use of SGAR compounds. This may be caused by the purchase and stock-piling of SGAR compounds prior to the restrictions, or the failure of the restrictions to reduce the risk of secondary exposure since eagles may be secondarily exposed to AR compounds through currently legal applications. The prevalence of exposure is concerning, and the documentation of SGAR toxicosis in eagles in this study suggests that exposure and mortality due to SGAR exposure remains a problem in eagles, despite recent risk mitigation efforts [23–25]. In addition to studying potential lethal doses and subclinical effects on individuals, continued monitoring of these compounds in eagles, other raptors, as well as mammalian carnivores or predators, may provide valuable insights into potential population-level impacts and identification of hotspots of exposure and mortality, both of which may influence future efforts to mitigate exposure.

## Supporting information

S1 Data (XLSX)

## References

[1] Grier JW (1982). Ban of DDT and subsequent recovery of reproduction in bald eagles.Science 218: 1232–1235. 10.1126/science.7146905 7146905

[2] Millsap BA, Zimmerman GS, Sauer JR, Nielson RM, Otto M, et al. (2013). Golden eagle population trends in the western United States: 1968–2010. J Wildl Manag 77: 1436–1448.

[3] Kirk DA, Hyslop C (1998). Population status and recent trends in Canadian raptors: a review. Biol Conserv 83:91–118.

[4] Erickson W, Urban D (Editors) (2004). Potential risks of nine rodenticides to birds and nontarget mammals: a comparative approach. Office of Prevention, Pesticides, and Toxic Substances. Office of Pesticides Programs, Environmental Fate and Effects Division. United States Environmental Protection Agency, Washington, D.C.

[5] Herring G, Eagles-Smith CA, Buck J (2017). Characterizing golden eagle risk to lead and antico-agulant rodenticide exposure: a review. J Raptor Res 51:273–292.

[6] Rattner BA, Lazarus RS, Elliot JE, Shore RF, van den Brink N. (2014). Adverse outcome pathway and risks of anticoagulant rodenticides to predatory wildlife. Environ Sci Tech 48: 8433–8445. 10.1021/es501740n 24968307

[7] Bachmann KA, Sullivan TJ (1983). Dispositional pharmacodynamics characteristics of brodifacoum in warfarin-sensitive rats. Pharmacology 27: 281–288. 10.1159/000137881 6657737

[8] Stone WB, Okoniewski JC, Stedelin JR (1999). Poisoning of wildlife with anticoagulant rodenticides in New York. J Wildl Dis 35: 187–193. 10.7589/0090-3558-35.2.187 10231745

[9] US Environmental Protection Agency (2008). Rodenticides Final Risk Mitigation Decision. Federal register: The Daily Journal of the US Government, 73 FR 31868, pp 31868–31869. https://www.federalregister.gov/documents/2008/06/04/E8-12493/rodenticides-final-risk-mitigation-decision-notice-of-availability. Accessed October 18, 2019.

[10] US Environmental Protection Agency, (2014). Product Cancellation Order for Certain Rodenticide Registrations. Federal Register: The Daily Journal of the US Government, 79 FR 45802, pp 45802–45803. https://www.federalregister.gov/documents/2014/08/06/2014-18361/product-cancellation-order-for-certain-rodenticide-registrations. Accessed October 18, 2019.

[11] Elliott JE, Hindmarch S, Albert CA, Emery J, Mineau P, et al. (2014). Exposure pathways of anticoagulant rodenticides to nontarget wildlife. Environ Monit Assess 186: 895–906. 10.1007/s10661-013-3422-x 24048882

[12] Murray M (2020). Continued anticoagulant rodenticide exposure of red-tailed hawks (Buteo jamaicensis) in the Northeastern United States with an evaluation of serum for biomonitoring. Environ Toxicol Chem 39(11):2325–2335. 10.1002/etc.4853 33405327

[13] Mendenhall VM, Pank LF (1980). Secondary poisoning of owls by anticoagulant rodenticides. Wildl Soc Bull 8: 311–315.

[14] Eason CT, Milne L, Potts M, Morriss G, Wright CRG et al. (1999). Secondary and tertiary poisoning risks associated with brodifacoum. N Z J Ecol 23: 219–224.

[15] Thomas P, Mineau P, Shore R, Champoux L, Martin P, et al. (2011). Second generation anticoagulant rodenticides in predatory birds: probabilistic characterization of toxic liver concentrations and implications for predatory bird populations in Canada. Environ Int 37: 914–920. 10.1016/j.envint.2011.03.010 21481471

[16] Christensen T, Lassen P, Elmeros M (2012). High exposure rates of anticoagulant rodenticides in predatory bird species in intensively managed landscapes in Denmark. Arch Environ Contam Toxicol 63: 437–444. 10.1007/s00244-012-9771-6 22588365

[17] Murray M (2011). Anticoagulant rodenticide exposure and toxicosis in four species of birds of prey presented to a wildlife clinic in Massachusetts, 2006–2010. J Zoo Wildl Med 42: 88–97. 10.1638/2010-0188.1 22946375

[18] Stansley W, Cummings M, Vudathala D, Murphy L (2014). Anticoagulant rodenticides in red-tailed hawks, Buteo jamaicensis, and great horned owls, Bubo virginianus, from New Jersey, USA, 2008–2010. Bull Environ Cont Toxicol 92: 6–9. 10.1007/s00128-013-1135-z 24158357

[19] Smith LL, Liang B, Booth MC, Filigenzi MS, Tkachenko A, et al. (2017). Development and val-idation of quantitative ultraperformance liquid chromatography-tandem mass spectrometry assay for anticoagulant rodenticides in liver. J Agric Food Chem 65: 6682–6691. 10.1021/acs.jafc.7b02280 28699743

[20] Ward MR, Stallknecht DE, Willis J, Conroy MJ, and Davidson WR(2006). Wild bird mortality and West Nile virus surveillance: biases associated with detection, reporting, and carcass persistence. J Wildl Dis 42: 92–106. 10.7589/0090-3558-42.1.92 16699152

[21] Quinn N. (2019). Assessing individual and population-level effects of anticoagulant rodenticides on wildlife. Hum-Wild Interact 13: 200–211.

[22] Hindmarch S, Elliot JE (2018). Ecological factors driving uptake of anticoagulant rodenticides in predators. In: van den Brink N, Elliott JE, Shore EF, Rattner BA, editors. Anticoagulant Rodenticides and Wildlife. Springer Nature, Cham, Switzerland, pp 228–258.

[23] Wayland M, Wilson LK, Elliott JE, Miller MJR, Bollinger T, et al. (2003). Mortality, morbidity, and lead poisoning of eagles in Western Canada, 1986–1998. J Raptor Res 37: 8–18.

[24] Harris MC, Sleeman JM (2007). Morbidity and mortality of bald eagles (Haliaeetus leucocepha-lus) and peregrine falcons (Falco peregrinus) admitted to the Wildlife Center of Virginia, 1993–2003. J Zoo Wildl Med 38: 62–66. 10.1638/05-099.1 17469277

[25] Russell RE, Franson JC (2014). Causes of mortality in eagles submitted to the National Wildlife Health Center 1975–2013. Wildl Soc Bull 38: 697–704.

[26] Hosea R. Exposure of non-target wildlife to anticoagulant rodenticides in California. In: Proceedings of the 19th Vertebrate Pest Conference; 2000 Mar 6–9; San Diego, CA. 2000. p. 236–44.

[27] Stone WB, Okoniewski JC, Stedelin JR (2003). Anticoagulant rodenticides and raptors: recent findings from New York, 1998–2001. Bull Environ Cont Toxicol 70: 34–40. 10.1007/s00128-002-0152-0 12478421

[28] Langford KH, Reid M, Thomas KV (2013). The occurrence of second generation anticoagulant rodenticides in non-target raptor species in Norway. Sci Total Environ 15: 205–208. 10.1016/j.scitotenv.2013.01.100 23500818

[29] Slankard KG, Gaskill CL, Cassone LM, Rhoden CM (2019). Changes detected in anticoagulant rodenticide exposure in barn owls (Tyto alba) in Kentucky, USA, in 2012–2016. J Wildl Dis 55: 432–437. 10.7589/2018-03-073 30289330

[30] Walker LA, Chaplow JS, Llewellyn NR, Pereira MG, Potter ED, et al. (2013). Anticoagulant rodenticides in predatory birds 2011: a predatory bird monitoring scheme (PBMS) report. Centre for Ecology and Hydrology, Lancaster, UK. 29 pp.

[31] Lambert O, Pouliquen H, Larhantec M, Thorin C, L’Hostis M. (2007). Exposure of raptors and waterbirds to anticoagulant rodenticides (difenacoum, bromadiolone, coumatetralyl, coumafen, brodifacoum): epidemiological survey in Loire Atlantique (France). Bull Environ Contam Toxicol 79: 91–94. 10.1007/s00128-007-9134-6 17487436

[32] Albert CA, Wilson LK, Mineau P, Trudeau S, Elliot JE (2010). Anticoagulant rodenticides in three owl species from western Canada, 1988–2003. Arch Environ Contam Toxicol 58: 451–459. 10.1007/s00244-009-9402-z 19826750

[33] Eason CT, Murphy E, Wright GR, Spurr EB (2002). Assessment of risks of brodifacoum to non-target birds and mammals in New Zealand. Ecotoxicology 11:35–48. 10.1023/a:1013793029831 11898799

[34] Rattner BA, Volker SF, Lankton JS, Bean TG, Lazarus RS, Horak KE (2020). Brodifacoum toxicity in American kestrels (Falco sparverius) with evidence of increased hazard on subsequent anticoagulant rodenticide exposure. Environ Toxicol Chem 39:468–481. 10.1002/etc.4629 31707739

[35] Bradbury S (Editor) (2008). Final risk mitigation decision for ten rodenticides. Office of Prevention, Pesticides, and Toxic Substances. United States Environmental Protection Agency, Washington, DC.

[36] Rattner BA and Harvey JJ. (2021). Challenges in the interpretation of anticoagulant rodenticide residues and toxicity in predatory and scavenging birds. Pest Manag Sci 77(2):604–610. 10.1002/ps.6137 33052019

[37] Hindmarch S, Rattner BA, Elliot JE (2019). Use of blood clotting assays to assess potential anti-coagulant rodenticide exposure and effects in free-ranging birds of prey. Sci Total Environ 657:1205–1216. 10.1016/j.scitotenv.2018.11.485 30677887

[38] Lemus JA, Bravo C, García-Montijano M, Palacin C, Ponce C, et al. (2011). Side effects of rodent control on non-target species: rodenticides increase parasite and pathogen burden in great bustards. Sci Total Environ 409: 4729–4734. 10.1016/j.scitotenv.2011.07.007 21889190

[39] Martínez-Padilla J, López-Idiaquez D, Lopez-Perea JJ, Mateo R, Paz A, et al. (2016). A negative association between bromadiolone exposure and nestling body condition in common kestrels: management implications for vole outbreaks. Pest Manag Sci 73: 364–370. 10.1002/ps.4435 27616006

