# Correction: Anticoagulant rodenticide exposure and toxicosis in bald eagles (*Haliaeetus leucocephalus*) and golden eagles (*Aquila chrysaetos*) in the United States

**DOI:** 10.1371/journal.pone.0328387

**Published:** 2025-07-15

**Authors:** Kevin D. Niedringhaus, Nicole M. Nemeth, Samantha Gibbs, Jared Zimmerman, Lisa Shender, Michelle van Deventer, Kate Slankard, Heather Fenton, Charlie Bahnson, Martha Frances Dalton, Elizabeth J. Elsmo, Robert Poppenga, Brian Millsap, Mark G. Ruder

Michelle van Deventer should be included in the author byline. Michelle van Deventer should be listed as the sixth author and affiliated with affiliation #3: Florida Fish and Wildlife Conservation Commission, Gainesville, FL, United States of America. The contributions of this author are as follows: Data curation, Funding acquisition, Investigation, Writing – review & editing.

The correct citation is: Niedringhaus KD, Nemeth NM, Gibbs S, Zimmerman J, Shender L, van Deventer M, Slankard K, et al. (2021) Anticoagulant rodenticide exposure and toxicosis in bald eagles (*Haliaeetus leucocephalus*) and golden eagles (*Aquila chrysaetos*) in the United States. PLoS ONE 16(4): e0246134. https://doi.org/10.1371/journal.pone.0246134.

The ninth author’s first and last name are switched. The correct name is: Charlie Bahnson.
